# Construction of a compound microbial agent for biocontrol against Fusarium wilt of banana

**DOI:** 10.3389/fmicb.2022.1066807

**Published:** 2022-12-20

**Authors:** Chanjuan Du, Di Yang, Yunfeng Ye, Lianfu Pan, Jin Zhang, Shangbo Jiang, Gang Fu

**Affiliations:** ^1^Key Laboratory of Green Prevention and Control on Fruits and Vegetables in South China Ministry of Agriculture and Rural Affairs, Guangxi Key Laboratory of Biology for Crop Diseases and Insect Pests, Plant Protection Research Institute, Guangxi Academy of Agricultural Sciences, Nanning, China; ^2^Horticultural Research Institute, Guangxi Academy of Agricultural Sciences, Nanning, China

**Keywords:** *Fusarium oxysporum* f. sp. *cubense*, combination agents, *Bacillus* sp., *Paenibacillus terrae*, *Trichoderma harzianum*

## Abstract

Banana wilt caused by *Fusarium oxysporum* f. sp. *cubense* has devastated a large number of banana plantations worldwide. Biological control is a possible method to conquer this disease. However, the control effect was often low and unstable while a single biocontrol strain had been applied in the field. Therefore, this study aimed to construct an effective compound microbial agent to control Fusarium wilt of banana (FWB) in the field. In addition to it, the compounding strategy of combining single strains for improving the control effect was investigated. Based on the compatibility test, five representative biocontrol strains were selected for the combination of all possible permutations. The pot experiment indicated that every biocontrol strain and their 26 combinations could control FWB to varying degrees. The control effect of combinations on FWB was higher than that of a single strain. In terms of the number of combinatorial biocontrol strains, the control effect of the four-strain combinations was the highest. According to the taxonomic differences of the five biocontrol strains, 26 biocontrol strain combinations could be divided into four groups. Among the strains in the combination, the larger the taxonomic differences the more easily it was to obtain a higher control effect. To obtain stable and efficient combinations, eight combinations were selected out and evaluated for their effectiveness in controlling FWB in different type soil. Compared with the other seven combinations, the four-strain combination T28 (Pt05 + Bc11 + Ba62 + gz-2) got the highest and stablest control effect in the four types of soil in greenhouse. And then the control effect of combination T28 was evaluated in field conditions, compared with commercially agents *Bacillus subtilis*, *Trichoderma harzianum*, and carbendazim. After four consecutive applications in the field, the control effect of T28 against FWB was the highest, reaching 57.14%. The results showed that combination T28 had a good application prospect, and the finding provided a reference for the construction of compound microbial agents.

## 1 Introduction

Banana (*Musa* spp.) is one of the most important fruit and food crops in tropical and subtropical regions worldwide (Dale et al., 2017). In 2018, the global banana planting area was about 5.73 million hectares, with an annual output of more than 115 million tons (FAO, 2020). Fusarium wilt of banana (FWB) caused by *Fusarium oxysporum* f. sp. *cubense* (*Foc*) has led to huge economic losses and seriously threatened the healthy development and safety of the global banana industry (Ploetz, 2006; Lin et al., 2008; Butler, 2013; Ghag et al., 2015). For a soil-borne disease, the control of FWB mainly depended on non-host crop rotation (Huang et al., 2012), chemical fungicide control (Nel et al., 2007), and breeding of disease-resistant varieties (Wang et al., 2022). However, FWB is particularly difficult to control. The reasons are as follows: (1) *Foc* can survive in the soil for more than 20 years, even in the absence of plant hosts (Stover, 1962; Buddenhagen, 2009); (2) chemical fungicides have difficulty killing *Foc* after soil application (Ploetz, 2015; Pegg et al., 2019); and (3) no commercial varieties are available with high *Foc* resistance and good agronomic traits (Bubici et al., 2019). Thus, it is urgent to explore new methods to control this disease (Ploetz, 2015; Pegg et al., 2019). Biocontrol microbes have been successfully applied in controlling soil-borne diseases, including peanut root rot (Sharma et al., 2012), Fusarium root rot in wheat (Wang et al., 2015), cumin wilt (Kumar et al., 2016), cucumber Fusarium wilt (Luo et al., 2019), and pepper root rot (Zhang et al., 2021). That provides new opportunities for FWB control.

Numerous biocontrol microbes, including *Pseudomonas* sp., *Xanthomonas* sp., *Bacillus* sp., *Burkholderia* sp., *Streptomyces* sp., *Trichoderma* sp., and *Rhizobium* sp., have been screened to prevent and control FWB (Raza et al., 2017; Duan et al., 2020; Wang et al., 2022). Although many biocontrol microbes were effective against *Foc in vitro* or in the greenhouse, their control efficiencies were often lower and unstable in the field, due to the single strain cannot cope with the variable environmental factors, such as soil pH, osmotic pressure, organic matter, and salt concentration (Denoth et al., 2002).

Compound microbial agents, which are composed of multiple biocontrol strains, have been shown to improve their control effect and stability against soil-borne diseases. For example, a compound agent composed of two arbuscular mycorrhizal fungi, *Trichoderma harzianum*, and *Pseudomonas fluorescens* was significantly effective than that of a single strain against tomato Fusarium wilt in the field (Srivastava et al., 2010). In another study, a combination of eight strains of *P*. *fluorescens* showed a higher control effect than a single strain in controlling tomato bacterial wilt caused by *Ralstonia solanacearum* (Hu et al., 2016). A combination consisting of seven bacteria strains had been used to control maize seedling blight caused by *Fusarium verticillioides*. The result indicated that the inhibited effects of each strain were not as strong as those of the combination (Niu et al., 2017). As we have seen, compound microbial agent may improve disease control effect compared to a single strain (Pandey and Maheshwari, 2007). However, the construction of compound microbial agents was often blind and random. There was a lack of systematic research on the compound strategy of biocontrol strains for improving control effect.

For the reasons above, this research aimed to: (1) construct an effective microbial agent to control FWB and (2) to investigate the combination strategy of synergy in biocontrol strains. Based on the compatibility test, representative strains were selected for the combination of all possible permutations, and their control effects on FWB were determined using a pot experiment. Moreover, to obtain combinations of biocontrol stains with stable and efficient control effects, the compound microbial agents were evaluated for their effectiveness in controlling FWB in various banana plantation soil. The control effect of the optimal combination was determined under field conditions. The results provide a reference for the effective prevention and control of FWB and the construction method of compound microbial agents.

## 2 Materials and methods

### 2.1 Fungal and bacterial strains

*Fusarium oxysporum* f. sp. *cubense* tropical race 4 (TR4, strain Foc1402) was isolated from banana plants infected with FWB from Wuming town, Nanning city, Guangxi province of China. Nine biocontrol strains were isolated from rhizosphere soil from bananas grown in Tanluo town, Nanning city, Guangxi province of China, including *Trichoderma harzianum* (strain gz-2), *Burkholderia cepacia* (strain Bc11), *Paenibacillus terrae* (strain Pt05), *Bacillus velezensis* (strain Blz02), *Bacillus amyloliquefaciens* (strain Ba02, Ba62, Ba63, Ba48, and Ba310). All fungi and bacteria were cultured on potato dextrose agar (PDA) and nutrient agar (NA), respectively, and maintained at the Plant Protection Research Institute, Guangxi Academy of Agricultural Sciences.

### 2.2 Biological agents and fungicides

*Bacillus subtilis* wettable powder (active ingredient content of 1 × 10^9^ bacteria cells^•^g^–1^) was purchased from the Redsun Group Co., Ltd. (Nanjing, China). *Trichoderma harzianum* water-soluble powder (active ingredient content of 1 × 10^9^ spores^•^g^–1^) was purchased from Moon (Guangzhou) Biotech Co., Ltd. (Guangzhou, China). Carbendazim wettable powder (active ingredient content of 50%) was purchased from Shanghai Yuelian Chemical Co., Ltd. (Shanghai, China).

### 2.3 Preparation of inocula

To prepare the *Foc* inoculum, fresh mycelia of strain Foc1402 was harvested from 7-day-old cultures grown on PDA medium, suspended in potato dextrose broth (PDB), and incubated at 150 r^•^min^–1^ and 28°C for 3 days. The spore suspension was then filtered to separate the mycelia and adjusted to a concentration of 1 × 10^6^ spores^•^mL^–1^ with sterile water.

To prepare a spore suspension of *T*. *harzianum* gz-2, fresh culture mycelia was scratched and suspended in PDB medium, then incubated at 150 r^•^min^–1^ and 28°C for 5 days. Spores of strain gz-2 were collected after filtering out the mycelium and adjusted to a final concentration of 1 × 10^8^ spores^•^mL^–1^ with sterile water.

Bacterial inocula were prepared as previously described (Zaim et al., 2013). The individual bacteria culture was inoculated in a nutrient broth (NB) medium, incubated at 150 r^•^min^–1^ and 28°C for 48 h. The suspension of each bacterium was adjusted with sterile water to a final concentration of 1 × 10^8^ bacteria cells^•^mL^–1^.

### 2.4 Compatibility test between bacterial strains and *Trichoderma harzianum*

To construct compound microbial agents, compatibility tests were conducted among nine biocontrol strains before they were combined, which were performed as described by Rajeela et al. (2018) and Mulaw et al. (2020), with a few modifications. In a sterile Petri dish (9 cm in diameter), three sterilized Oxford cups were vertically placed on the surface of 2% water agar (WA) medium. Each Oxford cup was arranged 2.5 cm apart from the other. One of the biocontrol strains was selected as the indicator strain. NA medium was inoculated with 1% (v/v) of the indicator strain suspension after cooling to 45°C. The mixture was rapidly mixed and immediately poured evenly onto WA plates containing Oxford cups. After the upper plate was completely solidified, the Oxford cups were removed with sterilized tweezers to form some small wells with a diameter of 6 mm. As the challenge strains, the remaining biocontrol strains were added to 20 μL of the previously prepared suspensions (1 × 10^8^ bacteria cells^•^mL^–1^ or 1 × 10^8^ spores^•^mL^–1^) in each well and incubated at 28°C for 48 h. For the blank control, the same amount of sterile water was added, and each treatment was repeated in triplicate. If a transparent inhibitory zone was observed around challenge strains, it was considered antagonism (+); otherwise, it was considered compatibility (–).

### 2.5 Control effect of biocontrol strains and their combinations on Fusarium wilt of banana

Banana seedlings (*Musa acuminata* L., AAA group, Cavendish subgroup, cv. “Williams”) susceptible to *Foc* TR4, which were purchased from Nanning Xiangjie Agricultural Technology Co., Ltd., in Guangxi, China, were used to determine the control effect of biocontrol strains and their combinations on FWB under greenhouse conditions. This study was performed as described by Saravanan et al. (2003) with a few modifications and was conducted from 24 June to 26 July 2020 in the greenhouse of the Plant Protection Institute of Guangxi Academy of Agricultural Sciences. Based on the results of the compatibility test, five compatible biocontrol strains were selected as representative strains, and combinations of all possible permutations were designed. Inocula of biocontrol strain combinations were prepared as follows: each of the biocontrol strains was cultured individually referring to section “2.3 Preparation of inocula” and mixed in an equal volume according to the combination design at a final concentration of 1 × 10^8^CFU^•^mL^–1^. Two-month-old banana seedlings were transplanted to plastic pots (20 cm diameter, 22 cm height) containing sterilized vermiculite. One seedling was transplanted to each pot and watered every second day. Ten days after transplanting, each seedling was inoculated with 100 mL of biocontrol strain inoculum. Twenty-four hours later, the seedlings were inoculated with 50 mL of *Foc* suspension (concentration 1 × 10^6^ spores^•^mL^–1^) after wounding. These seedlings continued to grow for 6 days and were again inoculated with the same volume of biocontrol strain inoculum. The biocontrol strains were applied twice during the experiment. As a control, 100 mL of sterilized NB medium was used instead of suspensions of biocontrol strains, and a total of 32 treatments were designed (Table 1). All treatments were repeated three times, and nine plants were used for each treatment. After being inoculated twice with biocontrol strains for 15 days, the corms of the banana seedlings were cut to detect the infection degree of *Foc*. The disease index was carried out based on the extent of corm discoloration on 0–7 grade: Grade 0 = No symptoms; Grade 1 = 1–25% initial corm discoloration; Grade 3 = 26–50% slight discoloration of the corm; Grade 5 = 51–75% discoloration of the corm; Grade 7 = over 76% complete discoloration of the corm (Orjeda, 1998). The disease index and control effect were calculated using the following formulas:


Disease⁢index

$$=\frac{\begin{array}{c} \Sigma (\mathrm{Number}\mathrm{of}\mathrm{diseased}\mathrm{plants}\mathrm{in}\mathrm{each}\mathrm{grade}\\ \times \mathrm{value}\mathrm{of}\mathrm{relative}\mathrm{grade}) \end{array}}{\begin{array}{c} \mathrm{total}\,\mathrm{number}\,\mathrm{of}\,\mathrm{plants}\,\mathrm{observed}\\ \times \mathrm{maximum}\,\mathrm{disease}\,\mathrm{grade} \end{array}}\times 100$$

Controleffect(%)

$$=\frac{\begin{array}{c} \mathrm{Disease}\,\mathrm{index}\,\mathrm{of}\,\mathrm{control}\,\mathrm{treatment}\\ -\mathrm{disease}\,\mathrm{index}\,\mathrm{of}\,\mathrm{biocontrol}\,\mathrm{agent}\,\mathrm{treatment} \end{array}}{\mathrm{disease}\,\mathrm{index}\,\mathrm{of}\,\mathrm{control}\,\mathrm{treatment}}\times 100\%$$

**TABLE 1:** Biocontrol strains and their combinations used to determine their biocontrol efficacy on Fusarium wilt of banana.

Number of strains	Treatments	Combination	Number of strains	Treatments	Combination
/	Control	N/A	Three	T16	Pt05 + Bc11 + Blz02
Single	T1	Pt05 alone		T17	Pt05 + Bc11 + Ba62
	T2	Bc11 alone		T18	Pt05 + Bc11 + gz-2
	T3	Blz02 alone		T19	Pt05 + Blz02 + Ba62
	T4	Ba62 alone		T20	Pt05 + Blz02 + gz-2
	T5	gz-2 alone		T21	Pt05 + Ba62 + gz-2
Two	T6	Pt05 + Bc11		T22	Bc11 + Blz02 + Ba62
	T7	Pt05 + Blz02		T23	Bc11 + Blz02 + gz-2
	T8	Pt05 + Ba62		T24	Bc11 + Ba62 + gz-2
	T9	Pt05 + gz-2		T25	Blz02 + Ba62 + gz-2
	T10	Bc11 + Blz02	Four	T26	Pt05 + Bc11 + Blz02 + Ba62
	T11	Bc11 + Ba62		T27	Pt05 + Bc11 + Blz02 + gz-2
	T12	Bc11 + gz-2		T28	Pt05 + Bc11 + Ba62 + gz-2
	T13	Blz02 + Ba62		T29	Pt05 + Blz02 + Ba62 + gz-2
	T14	Blz02 + gz-2		T30	Bc11 + Blz02 + Ba62 + gz-2
	T15	Ba62 + gz-2	Five	T31	Pt05 + Bc11 + Blz02 + Ba62 + gz-2

### 2.6 Stability of the control effect of biocontrol strain combinations

To obtain combinations of biocontrol stains with stable and efficient control effects, eight combinations with high biocontrol efficiency on FWB were selected to determine their control effect in different types of banana plantation soil. The experiment was conducted from 21 September to 23 October 2020 in the greenhouse of the Plant Protection Institute of Guangxi Academy of Agricultural Sciences. Soil was sampled from a 0–30 cm tillage layer in banana plantations of different regions in Guangxi Province: Type I (Luoxu town, Nanning city, N23°11′52″, E108°01′17″), Type II (Tanluo town, Nanning city, N22°57′17″, E107°54′22″), Type III (Natong town, Nanning city, N23°02′56″, E107°54′30″), and Type IV (Linfeng town, Baise city, N23°35′18″, E107°12′4″). The physicochemical properties of the soil are shown in Table 2. The soil sample was air-dried and sieved through a 2 mm sieve, then filled into fresh plastic pots (20 cm diameter, 22 cm height). Each type of soil sample was treated as follows: T9, T11, T14, T20, T25, T27, T28, and T29. As a control, the same volume of sterilized NB medium was used instead of biocontrol strain suspensions. All treatments were repeated three times, and nine plants were used for each treatment. This assay was conducted by referring to the method in section “2.5 Control effect of biocontrol strains and their combinations on Fusarium wilt of banana.”

**TABLE 2:** Physicochemical properties of soil samples from different banana plantations.

Soil type	Available *P* (mg/kg)	Available K (mg/kg)	Organic matter (g/kg)	pH	Effective boron (mg/kg)	Exchangeable Ca (cmol/kg)	Exchangeable Mg (cmol/kg)	Hydrolysable N (mg/kg)
Type I	34.6	901	23.4	4.1	0.42	2.2	0.4	817
Type II	45.0	167	29.3	4.2	0.29	0.8	0.1	151
Type III	49.9	434	28.2	4.5	0.52	4.0	0.4	136
Type IV	19.4	495	24.5	6.9	0.60	14.9	1.9	125

### 2.7 Field trial

The field trial was carried out on a banana plantation in Tanluo town, Nanning city, Guangxi province, China (N22°57′17″, E107°54′22″) from 27 May to 30 October 2021. The study site suffered severe serious FWB in the last year, with an incidence rate of 20–30%, and had been under continuous banana cropping for 5 years since 2016. The soil was clay loam in texture, with a pH of 4.2. The plot size was 0.24 hectares by 17 rows, and the plant spacing was 2.5 m between rows and 2 m within rows. There were 30 plants in each row. Banana plantlet (*Musa acuminata* L., AAA group, Cavendish subgroup, cv. Williams) was the perennial banana with 9–12 true leaves, and it was in the vegetative growth period during the treatments of biocontrol strain inoculum. There were four treatments as follows: (1) a biocontrol strain combination of T28 at a concentration of 1 × 10^9^ CFU^•^mL^–1^, applied at a rate of 62.5 L^•^ha^–1^. (2) *B*. *subtilis* wettable powder at a concentration of 1 × 10^9^ bacteria cells^•^g^–1^ (BD), applied at a rate of 62.5 Kg^•^ha^–1^; (3) *T*. *harzianum* water-soluble powder at a concentration of 1 × 10^9^ spores^•^g^–1^ (TD), applied at a rate of 62.5 Kg^•^ha^–1^; and (4) carbendazim wettable powder at a concentration of 50% (FD), applied at a rate of 420 g^•^ha^–1^. The same volume of water was used as the control treatment. Experiments were performed using a completely randomized design, and the two rows at the edge served as protective rows.

To prepare the biocontrol strain inoculum for field application, each member strain of T28 was activated according to the method described in section “2.3 Preparation of inocula.” The culture was inoculated into a 50-L fermenter (biotech-50JS, Shanghai Baoxing Biological Equipment Engineering Co., Ltd., Shanghai, China) containing a 10 L fermentation medium with 1% (*v/v*) inoculum for monoculture fermentation. The fermentation medium was the same as the activation medium for each strain. Strains Blz02 and Ba62 were fermented for 2 days at 150 rpm under aerobic conditions at 30°C, while strains Pt05 and gz-2 were fermented for 3 and 5 days, respectively. After fermentation was completed, the fermentation broth of each biocontrol strain was adjusted to a final concentration of 1 × 10^9^ CFU^•^mL^–1^ with sterile water and mixed with an equal volume before being applied in the field.

The field experiment was performed as described by Saravanan et al. (2003) and Damodaran et al. (2020), with a few modifications. The first inoculation was performed by spraying inoculum in the banana rhizosphere on 27 May 2021, with a volume of 0.33 L per plant used for field inoculation. Each treatment was repeated with three replicates, and 30 banana plants per replicate. Inoculation was performed four times in total at intervals of 1 month. Banana plants were managed by normal field practices during the period of the experiment without chemical fungicide or fertilizer. The number of infected plants in each treatment was counted on 30 October 2021, when the banana plants were in fruit-set periods, and the control effect (%) was calculated (*n* = 30) according to the following formula:


$$\mathrm{Control}\mathrm{effect}\left(\%\right)=\frac{\left(\mathrm{R1}-\mathrm{R2}\right)}{\mathrm{R1}}\times 100\%,$$

Where R1 and R2 indicate the number of infected plants in the control and treatment areas, respectively.

### 2.8 Statistical analysis

Statistical analyses of all data were carried out using SPSS software, version 18.0 (SPSS Inc., Chicago, IL, USA). Differences were subject to one-way analysis of variance (ANOVA) and Duncan’s multiple range test (DMRT) at *P* = 0.05. Differences at *P* < 0.05 were considered statistically significant. Each test was replicated three times. Charts were drawn using Origin 8.0 (Origin Lab, Massachusetts, USA).

## 3 Results

### 3.1 Compatibility among the biocontrol strains

**FIGURE 1:**
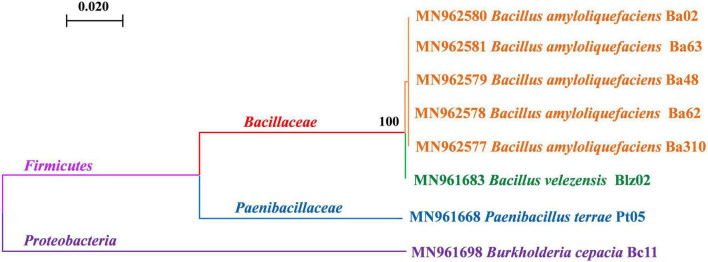
Evolutionary relationships of biocontrol bacteria. Phylogenetic analyses were conducted by maximum likelihood using the 16s rDNA sequences, which showed the genetic relationship between *Bacillus amyloliquefaciens*, *Bacillus velezensis*, *Paenibacillus terrae*, and *Burkholderia cepacia*. The numbers represent the accession numbers in GenBank. The confidence probability of each node was estimated using a bootstrap test (1,000 replicates). The scale bars represent a genetic distance of 0.02 substitutions per nucleotide position. All strains were labeled with different colors: orange was *B*. *amyloliquefaciens*, green was *B*. *velezensis*, blue was *P*. *terrae*, and purple was *B*. *cepacia*.

Nine biocontrol strains from two microbial kingdoms were used for the compatibility test, including one fungus (*T*. *harzianum* gz-2) and eight bacteria. A genetic kinship tree of the eight bacteria is shown in Figure 1. At the phylum level, these bacteria belonged to *Proteobacteria* (*B*. *cepacia* Bc11) and *Firmicutes*. Bacteria belonging to *Firmicutes* were divided into two families: *Paenibacillaceae* (*P*. *terrae* Pt05) and *Bacillaceae*. Among the six strains belonging to *Bacillus* sp., strain Blz02 was *B*. *velezensis* (strain Blz02), whereas the other five were *B*. *amyloliquefaciens* (strain Ba02, Ba62, Ba63, Ba48, and Ba310).

The results of the compatibility testing are shown in Table 3. Regardless of the indicator or challenge strain, *T*. *harzianum* gz-2 was compatible with the eight bacteria since there was no inhibition zone formed between them on the NA medium. Co-cultures of six *Bacillus* strains (*B*. *amyloliquefaciens* Ba02, Ba62, Ba63, Ba48, and Ba310 and *B*. *velezensis* Blz02) showed no inhibition zones against one another when they were used as the challenge strain, indicating that they were compatible. As a challenge strain, *P*. *terrae* Pt05 was compatible with all tested strains, but as an indicator strain, it was antagonistic to *B*. *amyloliquefaciens* Ba02, Ba63, Ba48, Ba310, and *B*. *cepacia* Bc11. Strain PT05 did not inhibit the growth of these bacteria but was inhibited by them when they were cultured together. Therefore, strains Pt05, Bc11, Blz02, Ba62, and gz-2, which were compatible with each other, were selected to construct a compound microbial agent.

**TABLE 3:** Compatibility assay among *Trichoderma* and bacterial strains.

Indicator strains	Challenge strains								
	gz-2	Bc11	Pt05	Blz02	Ba02	Ba62	Ba63	Ba48	Ba310
gz-2		–	–	–	–	–	–	–	–
Bc11	–		–	–	–	–	–	–	–
Pt05	–	**+**		–	**+**	–	**+**	**+**	**+**
Blz02	–	–	–		–	–	–	–	–
Ba02	–	–	–	–		–	–	–	–
Ba62	–	–	–	–	–		–	–	–
Ba63	–	–	–	–	–	–		–	–
Ba48	–	–	–	–	–	–	–		–
Ba310	–	–	–	–	–	–	–	–	

“+” indicates antagonism, and “−”indicates compatibility.

### 3.2 Efficiency of biocontrol strains and their combinations in controlling Fusarium wilt of banana

**FIGURE 2:**
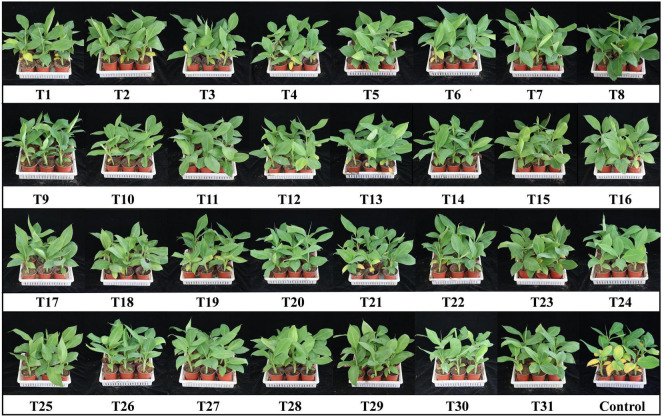
Symptoms of Fusarium wilt of banana observed on infected banana seedlings 15 days after inoculation.

**FIGURE 3:**
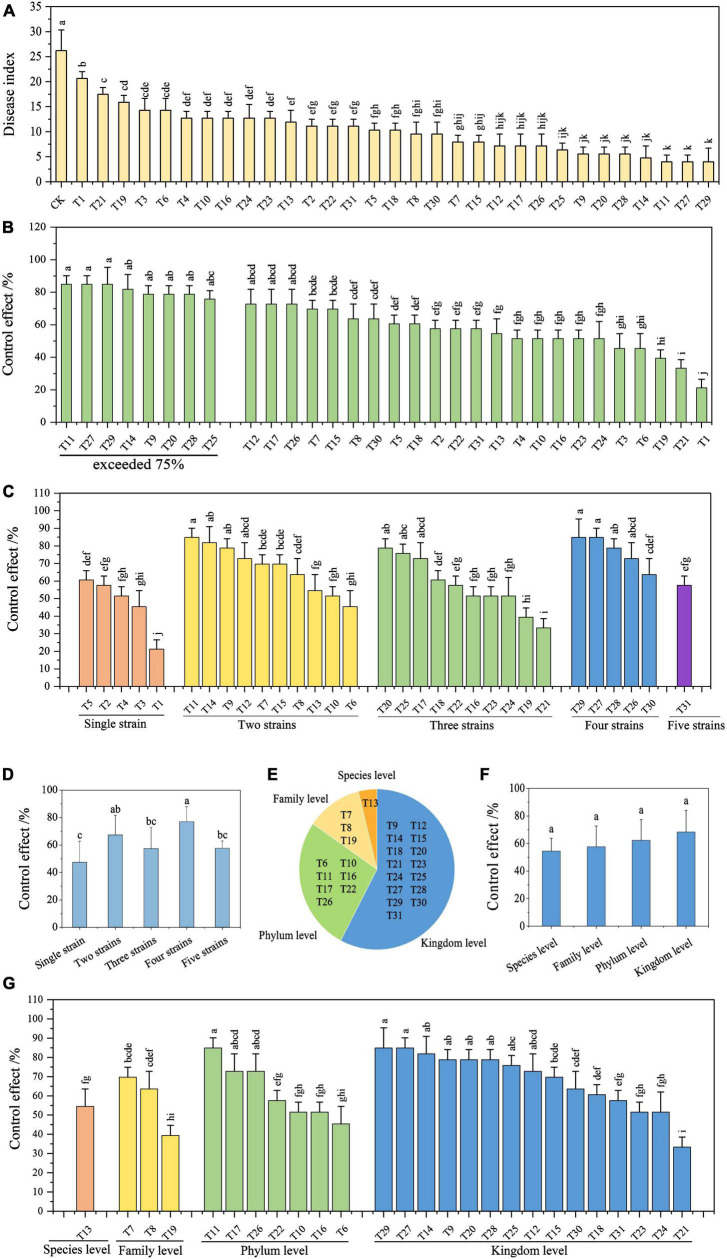
Efficiency of biocontrol strains and their combinations in controlling Fusarium wilt of banana. **(A)** Disease index of infected banana seedlings. **(B)** Control effect of each treatment on Fusarium wilt of banana. **(C)** Analysis of the control effect based on the strain number in the combinations. **(D)** Average control effect of combinations with a different number of strains. **(E)** Groups of biocontrol strain combinations based on genetic relationship differences. **(F)** Average control effect of combinations with genetic relationship differences. **(G)** Analysis of control effect based on genetic relationships in the combinations. Different lowercase letters above the bars denote significant differences among treatments (*P* < 0.05).

Fifteen days after the second inoculation, obvious symptoms started to appear in the control plants, while there were no visible symptoms in the treatment groups (Figure 2). Based on the results (Figure 3 and Table 4), the disease index of the control was the highest at 26.19, which was significantly higher than that of the biocontrol strains and ranged from 5.56 to 20.63. The results suggest that all biocontrol strains and their combinations significantly inhibited FWB (Figure 3A).

**TABLE 4:** Disease index of banana seedlings and control effect of biocontrol strains against Fusarium wilt of banana.

Treatments	Combination	Disease index	Control effect (%)
T1	Pt05 alone	20.63 ± 1.37 b	21.21 ± 5.25 j
T2	Bc11 alone	11.11 ± 1.37 efg	57.58 ± 5.25 efg
T3	Blz02 alone	14.29 ± 2.38 cde	45.45 ± 9.09 ghi
T4	Ba62 alone	12.70 ± 1.37 def	51.52 ± 5.25 fgh
T5	gz-2 alone	10.32 ± 1.37 fgh	60.61 ± 5.25 def
T6	Pt05 + Bc11	14.29 ± 2.38 cde	45.45 ± 9.09 ghi
T7	Pt05 + Blz02	7.94 ± 1.37 ghij	69.70 ± 5.25 bcde
T8	Pt05 + Ba62	9.52 ± 2.38 fghi	63.64 ± 9.09 cdef
T9	Pt05 + gz-2	5.56 ± 1.37 jk	78.79 ± 5.25 ab
T10	Bc11 + Blz02	12.70 ± 1.37 def	51.52 ± 5.25 fgh
T11	Bc11 + Ba62	3.97 ± 1.37 k	84.85 ± 5.25 a
T12	Bc11 + gz-2	7.14 ± 2.38 hijk	72.73 ± 9.09 abcd
T13	Blz02 + Ba62	11.90 ± 2.38 ef	54.55 ± 9.09 fg
T14	Blz02 + gz-2	4.76 ± 2.38 jk	81.82 ± 9.09 ab
T15	Ba62 + gz-2	7.94 ± 1.37 ghij	69.70 ± 5.25 bcde
T16	Pt05 + Bc11 + Blz02	12.70 ± 1.37 def	51.52 ± 5.25 fgh
T17	Pt05 + Bc11 + Ba62	7.14 ± 2.38 hijk	72.73 ± 9.09 abcd
T18	Pt05 + Bc11 + gz-2	10.32 ± 1.37 fgh	60.61 ± 5.25 def
T19	Pt05 + Blz02 + Ba62	15.87 ± 1.37 cd	39.39 ± 5.25 hi
T20	Pt05 + Blz02 + gz-2	5.56 ± 1.37 jk	78.79 ± 5.25 ab
T21	Pt05 + Ba62 + gz-2	17.46 ± 1.37 c	33.33 ± 5.25 i
T22	Bc11 + Blz02 + Ba62	11.11 ± 1.37 efg	57.58 ± 5.25 efg
T23	Bc11 + Blz02 + gz-2	12.70 ± 1.37 def	51.52 ± 5.25 fgh
T24	Bc11 + Ba62 + gz-2	12.70 ± 2.75 def	51.52 ± 10.5 fgh
T25	Blz02 + Ba62 + gz-2	6.35 ± 1.37 ijk	75.76 ± 5.25 abc
T26	Pt05 + Bc11 + Blz02 + Ba62	7.14 ± 2.38 hijk	72.73 ± 9.09 abcd
T27	Pt05 + Bc11 + Blz02 + gz-2	3.97 ± 1.37 k	84.85 ± 5.25 a
T28	Pt05 + Bc11 + Ba62 + gz-2	5.56 ± 1.37 jk	78.79 ± 5.25 ab
T29	Pt05 + Blz02 + Ba62 + gz-2	3.97 ± 2.75 k	84.85 ± 10.5 a
T30	Bc11 + Blz02 + Ba62 + gz-2	9.52 ± 2.38 fghi	63.64 ± 9.09 cdef
T31	Pt05 + Bc11 + Blz02 + Ba62 + gz-2	11.11 ± 1.37 efg	57.58 ± 5.25 efg
T32	Control	26.19 ± 4.12 a	–

Values are the means (± SEM) of the three replications. Means in a column with similar letter(s) were not significantly different (*P* < 0.05).

The control effect of a single control strain (T1–T5) on FWB ranged from 21.21 to 60.61%, with an average control effect of 47.27% (Figures 3C,D). Except for T6, T19, and T21, all control effects of the biocontrol strain combinations (T6–T31) were greater than 51.52% (Figures 3C,D). Of these, there were 11 combinations (T9, T11, T12, T14, T17, T20, T25, T26, T27, T28, and T29), with control effects exceeding 72.73%, and the highest was 84.85% (Figure 3B). Overall, the control effects of the compound microbial agents were better than those of a single strain.

Nevertheless, based on the number of strains in the combination, there was no positive relationship between the strain number and the control effect on FWB (Figure 3C). Four-strain combinations had the highest average control effect of 76.97%. The average control effects of the two-strain combinations and the three-strain combinations were comparable to those of the five-strain combination, which were 67.27, 57.27, and 57.58%, respectively (Figure 3D).

The 26 combinations of biocontrol strains were split into four groups based on their genetic relationships, with differences in species, family, phylum, and kingdom levels (Figure 3E). The average control effects of these four groups on FWB were 54.5, 57.58, 62.34, and 68.28%, respectively (Figures 3F,G). The more distantly related combinations tended to obtain higher control effects. Moreover, the combinations with the highest control effect appeared in distantly related groups at the phylum (T7, T11, and T26) and kingdom levels (T9, T12, T14, T20, T25, T27, T28, and T29) (Figure 3G). Eight combinations with a control effect exceeding 75% were selected to further evaluate their stability in controlling FWB (Figure 3B).

### 3.3 Effect of biocontrol strain combinations against Fusarium wilt of banana in different soil types

**FIGURE 4:**
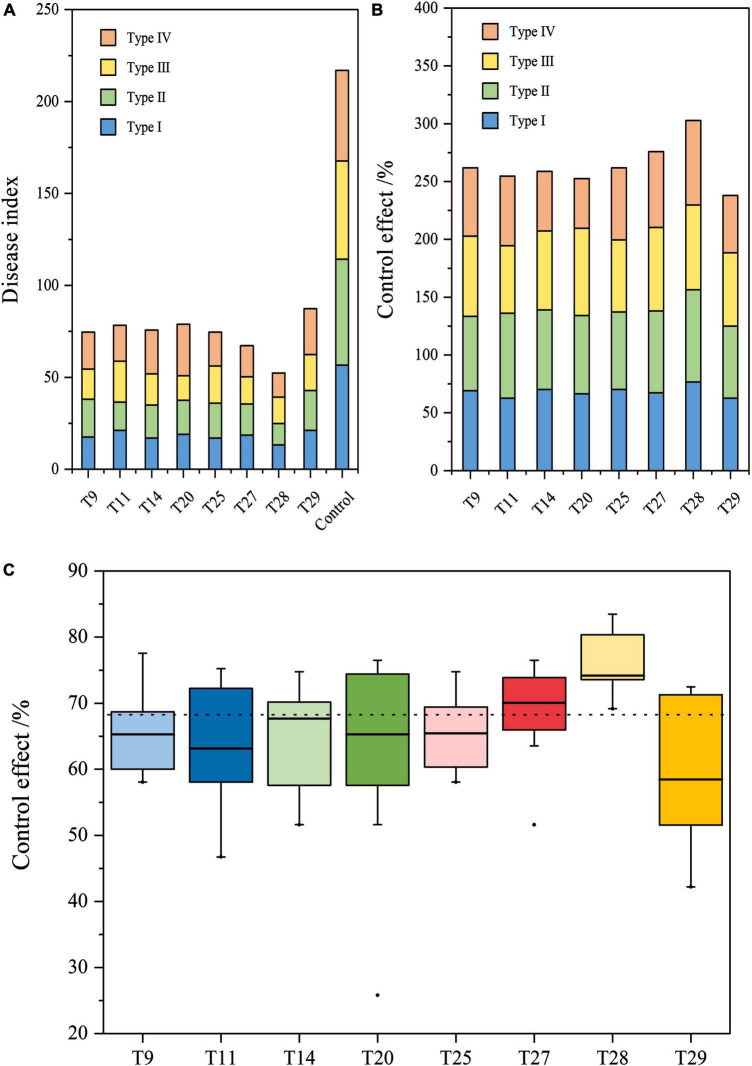
Efficiency of biocontrol strain combinations against Fusarium wilt of banana in different types of soil. **(A)** Disease index banana seedlings treated with the eight combinations. **(B)** Control effect of the eight combinations against Fusarium wilt of banana. **(C)** Stability of the control effect on the eight combinations. Boxplots depict minimum and maximum values (whiskers), the interquartile range (box), outliers (dots), and median (line).

The control effects of eight biocontrol strain combinations against FWB were determined in four types of soil. The disease index of all compound microbial agents was significantly lower compared with the control (*P* < 0.05). The disease index of compound microbial agents ranged from 11.64 to 28.04, while that of the controls ranged from 49.21 to 57.67 (Figure 4A). In different types of soil, the control effects of the compound microbial agents differed, ranging from 43.01 to 79.82% (Figure 4B). Of these, T28 showed the highest biocontrol efficiency against FWB, exceeding 73.12% in different soil types.

Regarding the stability of the control effect, T27 and T28 of the four-strain combination displayed the smallest magnitude of change in the four types of soil, while their median values were the highest at 68.95 and 75.71%, respectively (Figure 4C). The median values of all two- and three-strain combinations were between 63.13 and 65.46%, which were lower than those of T27 and T28. Their control effects varied greatly among the four types of soil. For all two- and three-strain combinations, their control effects varied greatly in the four types of soil, and were lower than those of T27 and T28, with median values ranging between 63.13 and 65.46%. Overall, T28 of the four-strain combination (Pt05 + Bc11 + Ba62 + gz-2) showed the best stability and control against FWB compared to the other 7 combinations in different types of soil.

### 3.4 Biocontrol efficiency of the T28 combination against Fusarium wilt of banana under field conditions

**FIGURE 5:**
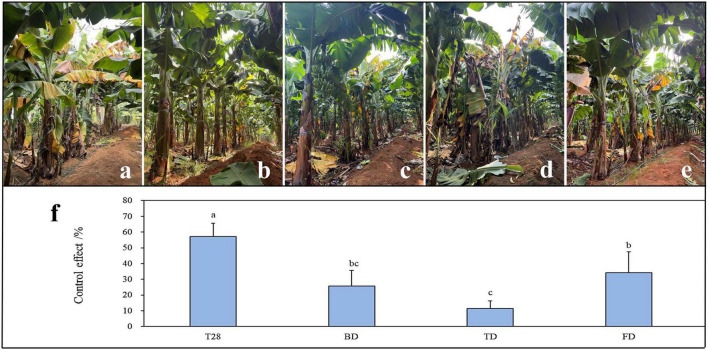
Control effect of different treatments against Fusarium wilt of banana in the field. **(a)** Control, **(b)** the biocontrol strain combination of T28 (T28), **(c)** *Bacillus subtilis* (BD), **(d)** *Trichoderma harzianum* (TD), and **(e)** Carbendazim (FD). **(f)** Data presented are the control effects of the four treatments against Fusarium wilt of banana after four consecutive applications. Values are the means (± SEM) of the three replications. Different lowercase letters above the bars denote significant differences among treatments (*P* < 0.05).

In comparison with commercial biological agents and fungicides, the control effect of the best combination T28 against FWB was determined under field conditions. After four consecutive applications in the field, significant differences were observed in the control effect between treatments (Figure 5). Compared to BD, TD, and FD treatments, T28 had a significantly higher control effect (*P* < 0.05), up to 57.14%. The control effect of TD was the lowest, at only 11.43%. In contrast, the control effects of BD were comparable to those of FD without any significant difference (*P* < 0.05).

## 4 Discussion

FWB is a devastating soil-borne disease in banana. Currently, no effective treatment is available for this disease. Biological control is considered an ideal way to control FWB. Many biocontrol microbes have been screened to control FWB, but their efficiency is unsatisfactory under field conditions (Getha et al., 2005). A single strain that is difficult to adapt to various soil environments might be one of the most important causes of poor efficiency. Therefore, many studies have attempted to use biocontrol strain combinations to control FWB. Different combinations of AM fungi, *T*. *harzianum* and *P*. *fluorescens*, were tested for controlling FWB, showing control effects of 40–80% in pot conditions and 42.85–64.29% in field conditions (Sukhada et al., 2010). Among the 11 combinations of *Trichoderma* sp. against FWB, Thangavelu and Gopi (2015) found that the combination of rhizosphere *Trichoderma* sp. nrcb3 and endophytic *T*. *asperellum* prr2 showed the best efficiency, with control effects of 100 and 54.90% in pot and field conditions, respectively. Gui et al. (2020) used a combination of non-pathogenic *F*. *oxysporum* sp., *Paecilomyce* sp., and *Trichoderma* sp. to control FWB under pot conditions and only found a control efficiency of 48%. In this study, we optimized the number and species of strains in the biocontrol strain combinations for controlling FWB. The highest combination efficiency was reached at 84.85% in the pot experiment, while the effect of the optimal combination T28 was 57.14% in the field.

Many studies have shown that the combination of biocontrol strains can significantly improve the effectiveness of disease prevention (Srivastava et al., 2010; Grosch et al., 2012; Alizadeh et al., 2013; Niu et al., 2017; Zaim et al., 2018; Izquierdo-García et al., 2020). Compared with similar results from these studies, most of the biocontrol strain combinations in this study were more effective in controlling FWB than single strains. Nevertheless, more strains in the combination do not always improve the disease control efficiency. In this study, the control effect of the five-strain combinations (T31) was not the highest among the 26 combinations tested, which was comparable to that of the single strain (T2, T3, T4, and T5). Four-strain combinations had the highest average control effect among all combinations. These results suggest that, in addition to the strain number and the defensive ability against the disease of each strain, the control effect of the combinations also depends on the interaction between the members (Whipps, 2001; Guetsky et al., 2002). The similar result was shown in the experiment that controlling FWB and root rot of papaya by combination of *Glomus mosseae*, *Trichoderma harzianum*, and *Pseudomonas fluorescens*. The best effects were both provided by *G. mosseae* + *T. harzianum*, but not the combination of all three strains (Sukhada et al., 2010, 2011). From the perspective of taxonomic differences between each strain in the combinations, among the 26 combinations tested, the combinations with the highest efficiency appeared at the phylum and kingdom levels, with an effect exceeding 72.73%. All combinations involving differences in species and family level showed control effects of less than 69.70%. Overall, the combinations containing fungi and bacteria had a higher control effect than the combinations containing only bacteria. This indicates that the further the genetic relationship in the taxonomy of the strains contained in the combination, the greater the potential of the combination to improve the control ability of the disease during the construction of a compound microbial agent. It means the diversity of strains is beneficial in improving disease prevention. Similar to this is that the high *Pseudomonas* diversity reduced pathogen density in the tomato rhizosphere and decreased the disease incidence of tomato bacterial wilt (Hu et al., 2016). The similar result was also illustrated in the experiment of controlling corn stalk rot with a compound bacterial agent composed of 7 different strains (Niu et al., 2017). This may occur because the biological characteristics of the strains in the combination are quite different due to their distant genetic relationships, leading to differences in disease-resistance mechanisms. Such a combination can provide a variety of disease-resistance mechanisms, a more stable community structure, and the occupation of complementary niches in complex environments to play a greater synergy role in disease control.

To imitate the field conditions, biocontrol strain combinations were evaluated for their ability and stability for controlling FWB in different types of banana plantation soil under pot conditions. Soil samples were collected from different regions of banana plantations, and their physicochemical properties differed (Table 1). The results showed that compared with the two- and three-strain combinations, a higher control effect, and better stability were more easily available for the four-strain combination. In particular, the four-strain combination T28 showed the most stable and highest control effect in different soils. T28 was composed of four different antagonistic microorganisms, which were *Paenibacillus terrae*, *Burkholderia cepacia*, *Bacillus amyloliquefaciens* and *Trichoderma harzianum*. The member strains in the combination are abundant in their genetic, ecological, and physiological diversity, may be resulting in their different colonization abilities in different types of soil. And the different strains may play a synergistic role in competing niches, regulating soil physicochemical properties, inducting defense enzymes of host, and influencing the function of rhizosphere microbial communities. This is conducive to formatting a healthy defense system in different soils, so that the disease can be controlled more effectively.

In this study, we determined the efficiency of biocontrol strains and their combinations in controlling FWB and evaluated the efficiency of stability in 11 combinations in different types of banana plantation soil under greenhouse conditions. Combination T28 was identified by its high control effect and strong stability against FWB, and its efficiency was evaluated under field conditions. However, in a complex soil environment, different combinations of biocontrol strains may exhibit different colonization abilities, whose interactions can be extremely complex. Through metagenomic and transcriptomic analysis, we can subsequently investigate the effects of compound microbial agents on the diversity and community structure function of the soil microorganisms around banana roots, mine their disease resistance-related genes, and explore the mechanisms of disease prevention and control. This can provide a theoretical basis for the biological control of FWB.

## Data availability statement

The original contributions presented in this study are included in the article/supplementary material, further inquiries can be directed to the corresponding author.

## Author contributions

CD performed the experiments, analyzed the data, and wrote the manuscript. DY and YY conducted the experiments and revised the manuscript. LP and JZ collected samples and conducted the experiments. SJ revised the manuscript. GF designed the experiments, supervised the project, and wrote the manuscript. All authors have read and agreed to the published version of the manuscript.
